# In Memoriam: Nicos Christofides (1942–2019)

**DOI:** 10.1007/s43069-021-00091-y

**Published:** 2022-02-24

**Authors:** Berç Rustem, Panos Parpas

**Affiliations:** Imperial College London, London, UK

Nicos Christofides came from Cyprus to study for his A levels in Folkestone. He had a passion for painting and for a while considered pursuing painting. In 1960, he won a scholarship to study Electrical Engineering at Imperial College and thus started his long association with Imperial. He graduated with a First Class Degree and subsequently completed his PhD in electrical engineering. He was awarded the Ferranti medal for his PhD work and had particularly fond memories of the ceremony as it was the first official event that he attended with his girlfriend and future wife, Ann.

The journal publication associated with his PhD thesis [1] was the topic of a discussion article in the Proceedings of the Institution of Electrical Engineers [2]. A quote from Dr. B. Adkins nicely captures the guiding principles of Nicos’s approach to research: “The paper is a very good example of how good results can be obtained by a combination of theoretical and experimental work – each side in fact supports the other”.

In 1968, Nicos joined as lecturer the Management Engineering Section of the Department of Mechanical Engineering at Imperial. This Management Engineering Section is the forerunner of the present Imperial College Business School. He started working on combinatorial optimization and graph theory problems. Along with the department head Professor Sam Eilon, he worked on vehicle routing algorithms. Nicos’ work on the travelling salesman problem is very well known. His worst-case performance guarantee heuristic algorithm for the travelling salesman problem [3] is groundbreaking and has collected over 2200 citations. It has never been published in an international journal until now. We are happy to have it published here in this issue as a tribute to Nicos. His vehicle routing software is still used today. Throughout his career, Nicos has been a pioneering researcher encompassing many combinatorial optimization areas, from theoretical results to the development of exact and heuristic algorithms for the effective solution of hard problems. His pioneering book Graph Theory: an Algorithmic Approach [4], published in 1975, has had an enormous impact not only in operational research, but also in economics and engineering. With characteristic humour, he used to joke that he liked to convert most problems into integer programming and then solve them.

Nicos became Professor of Operations Research in 1982 at Imperial College. During the 1980’s, he worked extensively on algorithms for image compression to allow images to be stored using a fraction of the memory that would be needed to store the actual image. He produced a successful system and worked extensively on improving its speed. He consulted for many industries, including oil and gas, telecoms as well as NASA and CDC in USA.

In 1990, Nicos established with Gerry Salkin the Centre for Quantitative Finance (CQF). The focus of CQF was to train PhD students in quantitative finance and risk management with a focus on real world problems. He developed a very effective forecasting system that was useful in his academic work as well as in his collaborations with industry and the finance sector. One of Nicos’ amazing achievements is that he has supervised in excess of 220 PhD students.

Nicos was excellent company. He had wide knowledge of art, politics, science, as well as food and drink. He was an excellent lecturer at conferences, with a unique ability for explaining mathematically complex problems and make extremely hard solutions seem easy. He used to take his academic responsibilities extremely seriously even after retirement. Nicos became Professor Emeritus of Quantitative Finance at Imperial College London, having retired from Imperial in 2009. He remained active after retirement but was deeply affected by the loss of his wife Ann in 2018. He passed away a year later. Those who knew him miss him greatly.
